# *PITX1* inhibits the growth and proliferation of melanoma cells through regulation of *SOX* family genes

**DOI:** 10.1038/s41598-021-97791-6

**Published:** 2021-09-15

**Authors:** Takahito Ohira, Suguru Nakagawa, Jumpei Takeshita, Hiroyuki Aburatani, Hiroyuki Kugoh

**Affiliations:** 1 Division of Genome and Cellular Function, Department of Molecular and Cellular Biology, Tottori University, 86 Nishi-Cho, Yonago, Tottori 683-8503 Japan; 2 Chromosome Engineering Research Center, Tottori University, 86 Nishi-Cho, Yonago, Tottori 683-8503 Japan; 3 Genome Science Division, Research Center for Advanced Science and Technologies, Tokyo University, 4-6-1 Komaba, Meguro-ku, Tokyo, 153-8904 Japan

**Keywords:** Cancer, Cell biology, Molecular biology, Oncology

## Abstract

Melanoma is one of the most aggressive types of cancer wherein resistance to treatment prevails. Therefore, it is important to discover novel molecular targets of melanoma progression as potential treatments. Here we show that paired-like homeodomain transcription factor 1 (*PITX1*) plays a crucial role in the inhibition of melanoma progression through regulation of SRY-box transcription factors (*SOX*) gene family mRNA transcription. Overexpression of *PITX1* in melanoma cell lines resulted in a reduction in cell proliferation and an increase in apoptosis. Additionally, analysis of protein levels revealed an antagonistic cross-regulation between SOX9 and SOX10. Interestingly, PITX1 binds to the *SOX9* promoter region as a positive regulatory transcription factor; *PITX1* mRNA expression levels were positively correlated with *SOX9* expression, and negatively correlated with *SOX10* expression in melanoma tissues. Furthermore, transcription of the long noncoding RNA (lncRNA), survival-associated mitochondrial melanoma-specific oncogenic noncoding RNA (*SAMMSON*), was decreased in *PITX1*-overexpressing cells. Taken together, the findings in this study indicate that *PITX1* may act as a negative regulatory factor in the development and progression of melanoma via direct targeting of the *SOX* signaling.

## Introduction

Melanoma is the most lethal form of skin cancer, and is one of the most aggressive types of human cancer that develops from melanocytes^1^. Surgical resection is the most common treatment for melanoma with early diagnosis, but the prognosis of highly metastasized melanoma is poor, with only a 5-year survival rate^2^. The aggressiveness of melanoma is attributed to the combined effects of oncogenic signal pathways and cancer-related transcription factors^3^. About 50% of all melanomas harbor activating *BRAF* mutations (over 90% V600E). *BRAF*^*V600E*^ has been implicated in different mechanisms underlying melanoma tumorigenesis, most of which are due to deregulated activation of mitogen activating protein kinase (MAPK) pathways^4^. Thus, *BRAF* inhibitors are important chemotherapeutic treatments for unresectable or metastatic *BRAF* mutated melanoma. Although the anti-tumor effects of *BRAF* inhibitors are impressive, the durability of the response is limited due to drug resistance^5^. However, immunotherapy is currently changing the landscape of melanoma treatment; immunomodulatory therapies, such as anti-PD-1 and anti-CTLA-4 antibody drugs, are the standard of care for patients with metastatic or unresectable melanoma. Unfortunately, despite the promising results, only 20–40% of the patients with melanoma show long-term benefits, whereas the remaining 80% develop resistance to these immune-checkpoint inhibitors^6^. Therefore, melanoma treatment requires the development of new targets that are effective against resistance.

Paired-like homeodomain 1 (*PITX1*), which belongs to the category of bicoid-related homeobox genes, plays a role in the development of the Rathke pouch and adult pituitary gland^7^. *PITX1* was originally found as a transcription factor gene with pituitary specific expression^8^. On the other hand, *PITX1* has multiple tumor suppressive functions that inhibit the *RAS* pathway^9^ and induce the activation of *p53* transcription^10^. In addition, we identified *PITX1* as a novel suppressor gene for human telomerase reverse transcriptase (*hTERT*), which mainly regulates telomerase, and is active in most tumors. PITX1 directly binds to specific PITX1 response element sites in the *hTERT* promoter region, resulting in telomerase inhibition^11^. Moreover, downregulation of *PITX1* is observed in various cancers including malignant melanoma^12–16^. Collectively, this evidence suggests that *PITX1* dysfunction induces activation of oncogenic pathways and promotes cancer development.

*SOX9* and *SOX10* in the SOX (SRY-box) gene family of transcription factors have a crucial role in neural crest (NC) development during the embryonic stage^17^. NC cells are a transient embryonic cell population that gives rise to most of the peripheral nervous system, chondrocytes and osteoblasts of craniofacial structures, and melanocytes. Previous studies demonstrated the essential role of *SOX10* in the pathogenesis of nevi and melanoma in both mice and humans by regulating cell proliferation and survival of melanocytic cells^18,19^. Additionally, mouse *Sox10* haploinsufficiency fully prevents *Nras*^*Q61K*^-driven formation of melanoma development in vivo, which shows that *SOX10* plays a crucial role in melanomagenesis^18^. In contrast, *SOX9* is expressed in normal human melanocytes but its expression is downregulated in nevi and melanoma. Moreover, overexpression of *SOX9* in both human and mouse melanoma cell lines inhibits cell proliferation in vitro and in vivo^20^. Intriguingly, another study showed that *SOX9* and *SOX10* are functionally antagonistic regulators of postnatal melanocyte and melanoma development. SOX9 protein suppresses *SOX10* transcription by directly binding to its promoter^21^. These findings indicate that downregulation of the *SOX9* could promote melanoma proliferation by activating *SOX10* expression. Alternatively, the overexpression of *SOX9* results in an increased invasion in vitro and increases the number of metastases in a mouse model^22^. Therefore, it is likely that *SOX9* displays oncogenic and tumor-suppressive functions, suggesting that *SOX9* plays a role in switching the phenotype of melanoma cells^23^.

Researchers have found that melanoma cells express two distinct gene expression signatures, and these signatures correlate with in vitro characteristics, which are reversible depending on their cellular microenvironments. One signature is characterized by the low expression of *SOX9* and high expression of *SOX10*. These melanoma cells are highly proliferative and less invasive in vitro; thus*,* they are named as proliferative phenotype. The other signature is characterized by the high expression of *SOX9* and low expression of *SOX10*. In contrast to the proliferative cells, these cells are highly invasive but have a low proliferative capacity in vitro and are named as invasive phenotype^21^. These findings suggest that the expression levels of *SOX9* and *SOX10* are a marker of melanoma subtype.

Survival Associated Mitochondrial Melanoma-Specific Oncogenic Non-Coding RNA (*SAMMSON*) was identified as being specifically expressed in melanoma by in silico analysis^24^. *SAMMSON* was expressed in most malignant melanomas but was barely detectable in normal melanocytes and benign lesions. *SAMMSON* is a target of the lineage-specific transcription factor *SOX10*. Importantly, *SAMMSON* silencing reduced melanoma cell growth and survival independently of the mutation state of *BRAF*, *NRAS*, or *p53*. Furthermore, *SAMMSON* silencing produced a response in *BRAF* inhibitor resistant melanoma cells and phenocopied the effects of downregulation of mitochondrial metabolism protein p32, causing dysfunction of mitochondria and resulting in tumor annihilation^24^. These findings show the potential of *SAMMSON* as an informative biomarker of malignancy and a novel therapeutic target of melanoma.

Here, we identified *PITX1* as a positive regulator of *SOX9* gene via direct binding to its promoter. Overexpression of *PITX1* in *SOX9*^low^/*SOX10*^high^-expressing human melanoma cell lines (proliferative phenotype) inhibited cell proliferation and tumor growth in xenografts, but that of SOX9^high^/SOX10^low^-expressing human melanoma cell lines (invasive phenotype) did not. Additionally, induction of *PITX1* strongly suppressed *SOX10* expression and *SAMMSON* transcription, which act as oncogenic driver genes in melanoma, via up-regulation of SOX9. Moreover, *PITX1* mRNA expression level was positively correlated with *SOX9* mRNA expression level in human melanoma and normal skin tissues. Taken together, these findings suggest that *PITX1* plays a suppressor role in the proliferative phenotype of melanoma cells as an upstream transcription factor of *SOX9* and *SOX10*.

## Results

### Transient overexpression of *PITX1* inhibits melanoma cell proliferation and induces apoptosis

**Figure 1:**
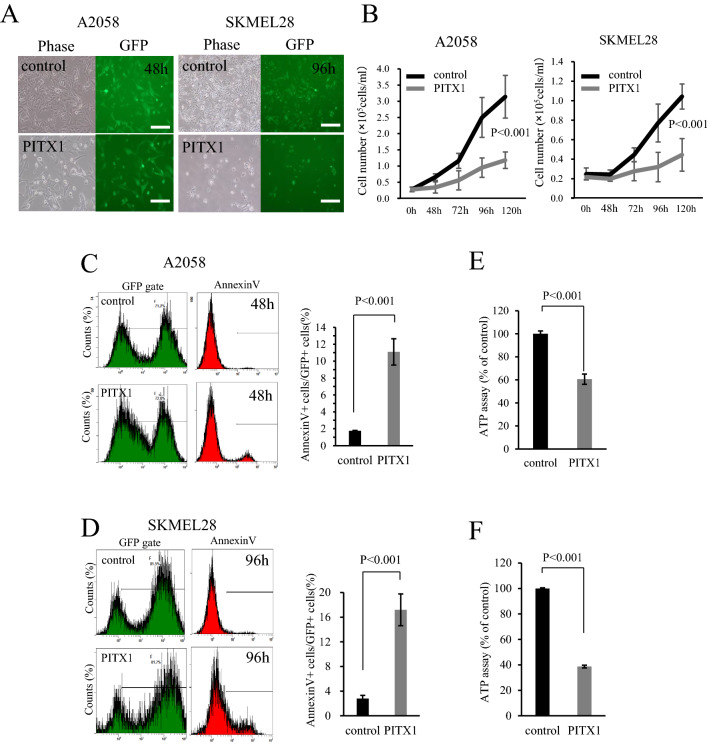
Overexpression of *PITX1* inhibits melanoma proliferation. (**A**) A2058 and SKMEL28 cell lines were infected with control and *PITX1* expression lentivirus vectors. Infection efficiency was monitored after 48 h or 96 h by fluorescence analysis of vector-encoded GFP (right panels). Phase contrast images are shown at left. Scale bars: 100 µm. (**B**) Number of *PITX1* transfected cells and control cells over 5 days. A2058 cells are shown at left, and SKMEL28 cells are shown at right. Bars correspond to means ± S.D. for three independent experiments (P < 0.001). Curve images of growth were created using Excel. (**C**,**D**) Flow cytometric analysis of apoptosis in *PITX1* overexpressing cells (**C**: A2058, **D**: SKMEL28). Quantification of apoptosis as % Annexin V positive cells in GFP positive gate. Bars correspond to means ± S.D. of three independent experiments (P < 0.001). Annexin V-positive cell count images were created using Kaluza software, and the bar graphs were created using Excel. (**E**,**F**) ATP assay in *PITX1*-overexpressing cells (**E**: A2058, **F**: SKMEL28). ATP content was measured using CellTiter-Glo 2.0 After Assay Kit at 96 h after infection with *PITX1* expressing or control lentivirus vector. Values are expressed as a percentage of control. Bars correspond to the means ± SD of three independent experiments (*P* < 0.001). These bar graphs were created using Excel.

We have previously identified that as a tumor suppressor gene, *PITX1* transcription factor directly regulates *TERT* expression in both mouse and human melanoma^11^. To investigate whether *PITX1* has multiple functions as a tumor suppressor gene, we generated A2058 (PITX1-A2058) and SKMEL28 (PITX1-SKMEL28) cells that transiently overexpress *PITX1* or a control vector. As shown in Fig. 1A, a fluorescence microscopy analysis 48 or 96 h after transfection indicated a high infection efficiency for both the control and *PITX1* lentiviral vector encoding *GFP*. The GFP-positive cell rate is shown in Supplementary Fig. S1. PITX1-A2058 and PITX1-SKMEL28 exhibited significantly decreased cell proliferation compared to control cells with the lentiviral vector (Fig. 1B). To further explore the effect of *PITX1* on cell proliferation, we analyzed apoptosis through flow cytometry using Annexin V staining and cytostasis through ATP assay. Annexin V positive cells gated on GFP had increased numbers of both PITX1-A2058 and PITX1-SKMEL28 cells compared to control cells (apoptotic cells in PITX1-A2058 were increased 6.6-fold, and 6.1-fold in PITX1-SKMEL28 compared to control cells, respectively) (Fig. 1C,D). Moreover, quantitative ATP levels were reduced in *PITX1*-induced cells (*PITX1*-A2058 and *PITX1*-SKMEL28 cells) compared with the control cells at 96 h (ATP level in *PITX1*-A2058 and PITX1-SKMEL28 was decreased by 40% and 60%, respectively, compared with the control cells) (Fig. 1E,F). These data indicate that overexpression of *PITX1* inhibited melanoma cell proliferation, accompanied by inducing apoptosis and cytostatic effect. On the other hand, reduction of telomerase activity through *PITX1* eventually leads to induction of a replication cellular senescence after several cell divisions. These results suggested that tumor suppression by *PITX1* may be functionally distinct from *TERT* repression.

### Identification of active enhancers in *PITX1*-expressing cells using ChIP-seq

**Figure 2:**
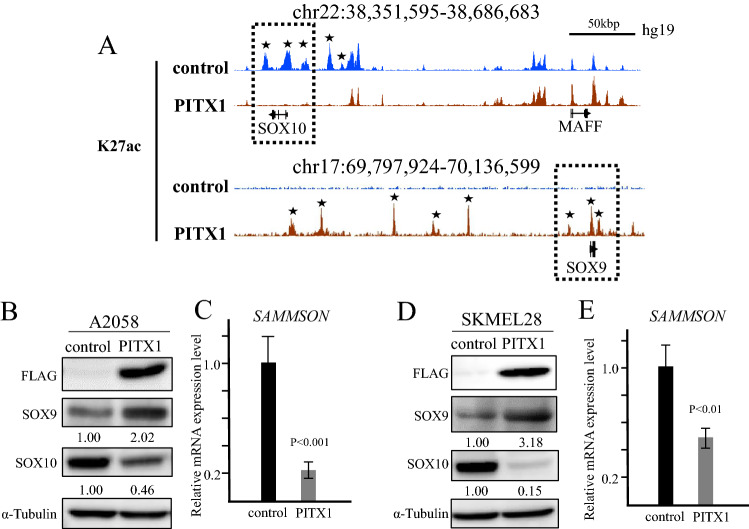
H3K27ac ChIP-seq in *PITX1*-expressing cells. (**A**) Integrated Genome Browser (IGB) screenshots of ChIP-seq data shows H3K27ac at the *SOX9* and *SOX10* loci in *PITX1* overexpressing A2058 (red) and control cells (blue). Stars indicates the regions where the peak of H27Kac differs between *PITX1*-overexpressing cells and control cells. (**B**) Western blotting analysis of the protein level of PITX1 (Flag tagged), SOX9 and SOX10 in A2058 cells at 48 h after infection with *PITX1* expressing or control lentivirus vector. The expression levels of SOX9 and SOX10 were normalized to α-tubulin levels. Cropped blots were used in this figure. Original full-length blots are presented in Supplementary Fig. S8. (**C**) qRT-PCR analysis of relative *SAMMSON* RNA expression levels in *PITX1* and control lentivirus vector infected A2058 cells. Expression in the vector control cells was arbitrarily set at 1. *GAPDH* mRNA expression was used as the internal control. Data are presented as means ± S.D. of three independent experiments (P < 0.001). (**D**) Western blotting analysis of the protein level of PITX1 (Flag tagged), SOX9 and SOX10 in SKMEL28 cells at 48 h after infection with *PITX1* expressing or control lentivirus vector. The expression levels of SOX9 and SOX10 were normalized to α-tubulin levels. Cropped blots were used in this figure. Original full-length blots are presented in Supplementary Fig. S8. (**E**) qRT-PCR analysis of relative *SAMMSON* RNA expression levels in *PITX1* and control lentivirus vector infected SKMEL28 cells. Expression in the vector control cells was arbitrarily set at 1. *GAPDH* mRNA expression was used as the internal control. Data are presented as means ± S.D. of three independent experiments (P < 0.01). All the bar graphs were created using Excel.

To determine target genes for *PITX1* in melanoma cells, we performed ChIP-seq analysis in *PITX1* stably transfected A2058 cells (PITX1s-A2058: Supplementary Fig. S2) using anti-Acetyl-Histone H3 Lys27 (H3K27ac) antibody. H3K27ac is one of the active enhancer markers in chromatin. We found that H3K27ac in PITX1s-A2058 cells, but not in control cells, was enriched across the SOX9 region, whereas the peaks around the *SOX10* genomic region disappeared. Conversely, MAF BZIP transcription factor F (*MAFF*), a gene encoding near *SOX10*, showed no change in the peaks around the gene region in these cells (Fig. 2A). To investigate whether the expression profiles for SOX9 and SOX10 are correlated with the change in status of H3K27ac, we performed a western blotting analysis of PITX1-A2058 cells using anti-SOX9 and SOX10 antibodies. As shown in Fig. 2B, SOX9 and SOX10 expression levels were significantly increased and decreased in PITX1-A2058 cells, respectively. Interestingly, the expression of *SAMMSON*, which is known as a melanoma-specific long non-coding RNA that is regulated by *SOX10*^24^, coincided with a reduction in SOX10 (Fig. 2C). In addition, a similar phenomenon was also observed in PITX1-SKMEL28 cells (Fig. 2D,E). These results suggest that *PITX1* could be a vitally important upstream regulator that controls the signaling pathway connecting *SOX9* to *SAMMSON *via* SOX10*.

A2058 and SKMEL28 cells are categorized in the proliferative phenotype of melanoma cells (*SOX9*^high^/*SOX10*^low^). To investigate whether the inhibition of growth by *PITX1* was also observed in the invasive phenotype of melanoma cell lines (*SOX9*^high^/*SOX10*^low^), we performed overexpression analysis of *PITX1* in the WM3211 melanoma cell line^25^. Western blotting showed that WM3211 cells expressed *SOX9* at a higher level than A2058. However, the expression level of *SOX10* was low in WM3211 cells (Supplementary Fig. S2A). Overexpression of *PITX1* in WM3211 did not significantly affect cell proliferation compared with the control cells (Supplementary Fig. S2B,C). Additionally, *SOX9* and *SOX10* protein levels indicated no change after inducing *PITX1* (Supplementary Fig. S2D). These results suggest that the inhibition of cell proliferation by *PITX1* is induced in the proliferative phenotype of melanoma cells but not in the invasive phenotype of melanoma cells.

### PITX1 directly binds to the *SOX9* promoter in vivo

**Figure 3:**
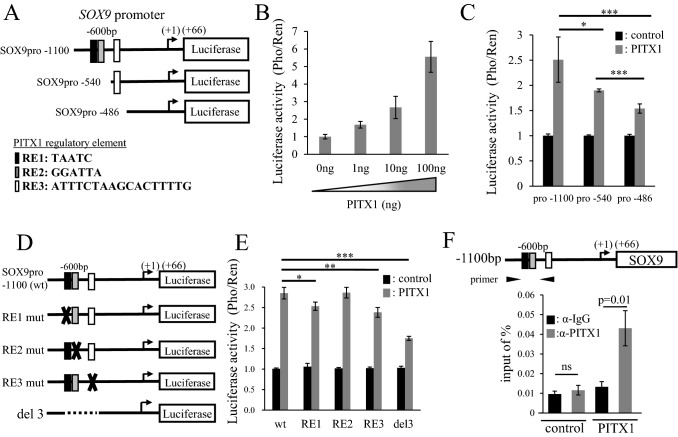
Upregulation of *SOX9* promoter transcriptional activity by PITX1. (**A**) The schematic drawing shows the position of the truncation site of each *SOX9* reporter plasmid. Black, gray and white boxes indicate *PITX1* regulatory elements (RE). (**B**) *PITX1* enhanced relative luciferase activity in a dose-dependent manner. The firefly luciferase activity (Pho) was standardized using *Renilla reniformis* luciferase activity (Ren) from co-transfected pGL4.70. Luciferase activity in empty-vector-transfected cells (PITX1:0ng) was arbitrarily set at 1. Data are presented as means ± S.D. for three independent experiments. (**C**) *PITX1* expression vectors were co-transfected into the human melanoma cell line A2058 with reporter plasmids containing a full-length promoter (SOX9pro − 1100), or a truncated *SOX9* promoter region in which RE sites in the *SOX9* promoter region partly or fully eliminated (SOX9pro − 540 and SOX9pro − 486). Luciferase activity in empty-vector-transfected cells was arbitrarily set at 1. Data are presented as means ± S.D. for three independent experiments (*P < 0.05, ***P < 0.001). (**D**) The schematic diagram shows the luciferase reporter plasmids that encode wild-type and RE mutated versions of the *SOX9* promoter region (RE1 mut, RE2 mut and RE3 mut). The cross mark represents mutation. The del3 reporter plasmids are deleted of RE1, RE2 and RE3 in the *SOX9* promoter region. (**E**) *PITX1* expression vectors were co-transfected with reporter plasmids containing the wild-type, RE-mutated, or deleted versions of the *SOX9* promoter. Luciferase activity in empty-vector-transfected cells was arbitrarily set at 1. Data are presented as means ± S.D. for three independent experiments (*P < 0.05, **P < 0.01, ***P < 0.001). (**F**) Schematic diagram showing the *SOX9* promoter and three boxes indicating PITX1 binding regions (upper panel). The black arrows indicate qPCR primers for the RE region in the *SOX9* promoter based on a ChIP assay. ChIP assay of RE regions in the *SOX9* promoter, showing enrichment with PITX1 antibody compared with IgG controls in A2058 clones. Anti-IgG antibody was used as a negative control. Input represents qPCR for the *SOX9* promoter DNA before immunoprecipitation. The data represent the ratio of the target fragment to the input DNA. Bars correspond to means ± S.D. for three independent experiments (P = 0.01, ns: not significant). All the bar graphs were created using Excel.

To investigate whether *PITX1* upregulates *SOX9* mRNA expression through modulation of *SOX9* promoter activity, we constructed a *SOX9* promoter-luciferase reporter plasmid (pGL3) containing a 1100-bp fragment (SOX9pro-1100) within the *SOX9* promoter region (Fig. 3A). Further, to identify potential PITX1 regulatory elements (RE) within the *SOX9* gene promoter, we performed in silico analysis using the TRAP web tool^26^. As a result, we identified three RE sites within the *SOX9* promoter. The sites/sequences in the *SOX9* promoter region are RE1 (TAATC: − 592/− 588), RE2 (GGATTA: − 578/− 573) and RE3 (ATTTCTAAGCACTTTTG: − 520/− 504) (Fig. 3A and Supplementary Fig. S4). We then investigated the effect of *PITX1* co-transfection with the SOX9pro − 1100 Luc reporter into A2058 cells on the transcriptional activity of the *SOX9* promoter by measuring Luc reporter activity. The result showed that *SOX9* promoter activity was increased by *PITX1* overexpression in a dose-dependent manner (Fig. 3B). We next constructed various truncated fragments of the 5′ region of the *SOX9* gene (SOX9pro − 540 reporter plasmid including only RE3, and SOX9pro − 486 reporter plasmid without RE) to identify the functional element in RE3 (Fig. 3A). Although SOX9pro − 1100 yielded the strongest promoter activity of these constructs, the promoter activities of SOX9pro − 540 and SOX9pro − 486 were decreased by 30% and 40%, respectively, compared to SOX9pro − 1100. These results suggested that critical positive regulatory elements are present between the − 1100 and − 486 regions. To further explore important REs in the *SOX9* promoter, we generated *SOX9* mutant promoter vectors containing the RE1 mut (altered TAATC to GTACC), RE2 mut (altered GGATTA to GGTACC), and RE3 mut (altered ATTTCTAAGCACTTTTG to GGTACCGGTACCCAGG) sequences (Fig. 3D). *SOX9* promoter activity for the mutation of RE1 and RE3, but not RE2, was reduced by approximately 12% compared to SOX9pro − 1100 (wt) (Fig. 3E). We next constructed reporter plasmids with deletion of RE1, RE2 and RE3 in the *SOX9* promoter region (del3) (Fig. 3D). SOX9 promoter activity decreased by 40% in del3 compared to parental SOX9pro − 1100 (Fig. 3E), and showed the same level as that of the truncated fragment SOX9pro − 486 (Fig. 3C). This result suggested that PITX1 could directly access RE1 and RE3 in the *SOX9* promoter region.

To determine whether PITX1 directly binds to the *SOX9* promoter, we performed a ChIP assay using nuclear extracts prepared from PITX1s-A2058 cells and control cells. The promoter region containing RE sites in the *SOX9* promoter exhibited fourfold amplification (qPCR) from chromatin that was precipitated with the anti-PITX1 antibody from PITX1 expressing cells, compared to control cells (Fig. 3F). These findings suggest that PITX1 can directly bind to REs in the *SOX9* promoter in A2058 cells.

### Expression profile of *PITX1*, *SOX9* and *SOX10* in human melanoma tissue

**Figure 4:**
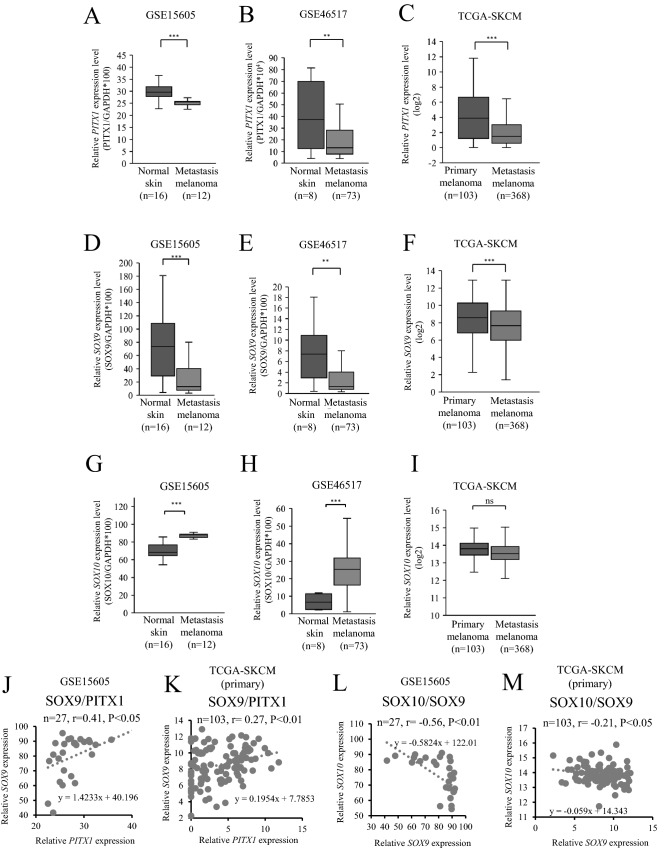
*PITX1* and *SOX9* are positively correlated in clinical melanoma tissues. (**A**–**I**) The relative expression levels of *PITX1*, *SOX9,* and *SOX10* in metastatic melanoma samples and normal skin or in primary and metastasis melanoma samples were determined using the GSE15605, GSE46517, and TCGA-SKCM RNA-seq datasets. The expression levels were normalized to *GAPDH* in GEO dataset (**P < 0.01, ***P < 0.001). The expression levels showed log2 scale in the TCGA dataset (***P < 0.001, ns: not significant). (**J**) Relationship between *PITX1* mRNA expression level and *SOX9* mRNA expression level in metastatic melanoma samples and normal skin. Pearson’s correlation coefficient (r) was calculated for *PITX1* and *SOX9* mRNA expression levels in the sixteen normal skin and twelve metastatic melanoma samples. (**K**) Relationship between *PITX1* and *SOX9* mRNA expression levels in metastatic melanoma samples and normal skin. Pearson’s correlation coefficient (r) was calculated for *PITX1* and *SOX9* mRNA expression levels in 103 primary melanoma samples. (**L**) Relationship between *SOX10* mRNA expression level and *SOX9* mRNA expression level in metastatic melanoma samples and normal skin. Pearson’s correlation coefficient (r) was calculated for *SOX10* and *SOX9* mRNA expression levels in the sixteen normal skin and twelve metastatic melanoma samples. (**M**) Relationship between *SOX10* and *SOX9* mRNA expression levels in metastatic melanoma samples and normal skin. Pearson’s correlation coefficient (r) was calculated for *SOX10* and *SOX9* mRNA expression levels in 103 primary melanoma samples. Box and scatter plots were created using Excel.

To further investigate the relationship between *PITX1*, *SOX9* and *SOX10* expression and clinical prognosis, we used RNA-seq datasets (GSE46517, GSE15606, and the cancer genome atlas [TCGA]-SKCM) from the Gene Expression Omnibus (GEO) and TCGA database to measure the expression level of these genes in melanoma primary, metastatic, and normal skin tissue. As shown in Fig. 4A,B, the relative expression of *PITX1* was lower in metastatic tissue compared to normal skin tissue. Additionally, *PITX1* expression was lower in metastatic tissue than in primary melanoma tissue (Fig. 4C). Similarly, *SOX9* expression was lower in metastatic tissue compared to normal skin tissue (Fig. 4D,E). Moreover, the relative expression of *SOX9* was lower in metastatic tissue than in primary melanoma tissue (Fig. 4F). In contrast, *SOX10* expression was higher in the metastatic tissue compared to normal skin tissue (Fig. 4G,H). However, there was no sufficient difference in *SOX10* expression between primary and metastasis melanoma tissue (Fig. 4I). A possible reason is that *SOX9*/*SOX10* signaling may be disrupted in highly malignant melanoma cells; therefore, it likely plays a more important role in carcinogenesis compared with that in malignancy. Furthermore, in agreement with our result, *PITX1* expression levels were positively correlated with *SOX9* levels in tumor and normal skin tissues (Fig. 4J). In primary melanoma tissue, we found a positive correlation between *SOX9* and *PITX1* expression in the TCGA data set (Fig. 4K). According to a previous report^21^, *SOX9* expression levels were negatively correlated with *SOX10* levels in tumor and normal skin tissues (Fig. 4L). Expression levels of *SOX9* were negatively correlated with those of *SOX10* levels in the TCGA primary melanoma tissue data set; however, it is lower than the GEO data set (Fig. 4M). These findings suggested that a loss of *PITX1* expression leads to up-regulation of *SOX10* by down-regulation of *SOX9,* eventually promoting the progression of human melanoma tissue.

### Gene activation of *PITX1* inhibits melanoma proliferation in vivo

**Figure 5:**
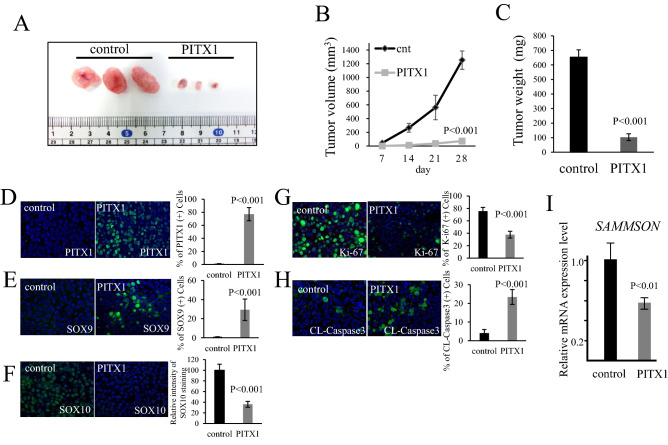
*PITX1* inhibits tumor growth in vivo. (**A**) Photograph of subcutaneous tumors generated in Balb-c/nu/nu mice with PITX1 transfected A2058 cells and that of the control. The left shows the control (n = 3), and the right shows PITX1 (n = 3). (**B**,**C**) Quantification of tumor volume and weight in *PITX1* and control samples. The tumor volume curve and bar graph for tumor weight were created using Excel. (**D**–**H**) Immunostaining of paraffin sections of the subcutaneous tumors for PITX1, SOX9, SOX10, Ki-67 and cleaved caspase-3 (FITC: Green). DAPI staining for nuclei (blue). Merged FITC and DAPI fluorescent images are shown. Each individual channel’s image is shown in Supplementary Fig. S4. Bar graph shows the number of positive expressing cells (or relative intensity level) for each gene in the *PITX1* sample compared to control. Data are presented as means ± S.D. for three independent experiments (P < 0.001). Scale bars: 100 µm. (**I**) qRT-PCR analysis of relative *SAMMSON* RNA expression levels in the subcutaneous tumors. Control cells were arbitrarily set as 1. *GAPDH* mRNA expression was used as the internal control. Data are presented as means ± S.D. for three independent experiments (P < 0.01).

To determine whether *PITX1* is important for melanoma growth in vivo, we performed subcutaneous tumor growth analysis using PITX1s-A2058 cells. A2058 cells with *PITX1* activation showed significantly smaller tumors than control cells in Bulb-c/nu/nu mice (Fig. 5A). The tumor volume with PITX1s-A2058 was remarkably decreased to 10% of that for the control cells 28 days after subcutaneous inoculation (Fig. 5B). Moreover, the tumor weight was decreased to 15.7% compared to the control cells (Fig. 5C). These results indicate that *PITX1* has inhibitory effects on melanoma growth in vivo.

**Figure 6:**
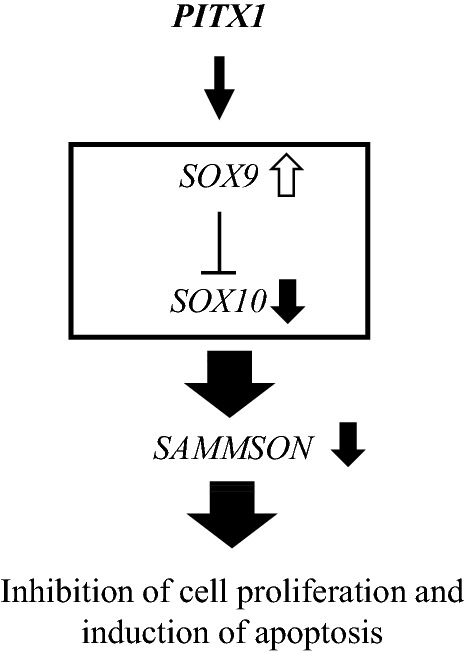
Working model for *PITX1* function in melanoma. *PITX1* activates *SOX9*, which suppresses *SOX10* and *SAMMSON* to inhibit melanoma growth and proliferation.

To investigate the gene expression profile of tumors produced from PITX1s-A2058 cells, we performed immunostaining analysis using anti-PITX1, SOX9, SOX10 Ki-67 and cleaved Caspase3 (CL-Caspase3) antibodies. PITX1 and SOX9 showed markedly increased levels in tumors stably expressing PITX1 (Fig. 5D,E). In contrast, SOX10 expression was reduced in this tumor tissue (Fig. 5F). We confirmed these results using qRT-PCR and western blotting (Supplementary Fig. S6). Furthermore, the expression of Ki-67 was significantly reduced in this tumor tissue (Fig. 5G). Conversely, the expression level of CL-Caspase3, which is a known apoptosis marker, was remarkably increased in the same tumor tissue (Fig. 5H). Overexpression of *PITX1* induced apoptosis in 23% of the cells in vivo, but this is insufficient to explain the inhibitory effect on melanoma growth (Fig. 5B). Cytostasis may also be thought to be induced by *PITX1* expression (Fig. 1E). Intriguingly, downregulation of SOX10 expression in tumor cells was associated with decreased *SAMMSON* expression, suggesting that *PITX1* plays a crucial role as a regulatory factor to directly suppress melanoma growth thorough the *SOX9-SOX10* and *SAMMSON* pathway (Fig. 5I). The findings of this study revealed that *PITX1* has a multifunctional role in tumor suppression, such as in the regulation of telomerase activity and the *SOX* signaling (Fig. 6).

## Discussion

We have reported that *PITX1* directly regulated *SOX9* through targeting the promoter region in melanoma cells. Intriguingly, increased *SOX9* expression by *PITX1* eventually leads to suppressive effects on cell proliferation and induction of apoptosis via downregulation of *SOX10* and *SAMMSON,* which play an oncogenic role in melanoma. Thus, our findings in this study provide evidence that *PITX1* has the ability to act as an upstream regulator of the *SOX9-SOX10* and *SAMMSON* pathways. Interestingly, another group showed that *PITX1* activates the *SOX9* promoter through a unique binding motif during human astrocyte differentiation^27^. This suggests that *PITX1* functions as an upstream factor of *SOX9* during physiological differentiation in normal cells.

Although upregulation of *SOX9* expression in *SOX10*-inhibited melanoma cells was observed as a reduction in cell proliferation and metastasis, high levels of *SOX9* expression can restore melanoma progression without an effect on *SOX10*^28^, suggesting that this different phenotype depends on the *SOX9* expression level. High levels of *SOX9* expression induce up-regulation of *NEDD9* (Neural Precursor Cell Expressed, Developmentally Down-Regulated 9), a member of the Crk-associated substrate (CAS) family of signal transduction proteins, instead of *SOX10* inhibition. *NEDD9* has a role as a scaffolding protein in regulating tumor progression in various cancers including melanoma^28^. In our study, *NEDD9* expression was not affected by the overexpression of *PITX1* (Supplementary Fig. S7). This data suggested that the control mechanism of *SOX9* by *PITX1* is different from the regulator of the SOX9-NEDD9 signaling pathway that causes the development of melanoma cells.

In this study, we demonstrated the inhibitory effect of *PITX1* in the proliferative phenotype of melanoma cell lines. In contrast, RNA-seq data comparing proliferative and invasive melanoma samples showed that the expression of *PITX1* and *SOX9* was upregulated in the invasive state^23^. Therefore, a detailed analysis of the role of *PITX1* in the invasive phenotype of melanoma cells is an important issue to be addressed in the future.

We previously found that microRNA-19b (miR-19b) directly inhibits *PITX1* mRNA translation through a miR-19b binding site within the 3′ UTR of *PITX1* mRNA^29^. Moreover, the expression of miR-19b depends on the *SOX10* expression level in Schwann cells, whose precursors arise from the neural crest. Indeed, reduction of *SOX10* expression by siRNA induced a decrease in miR-19b expression^30^. These results show that *SOX10* overexpression may trigger an increase in the levels of miR-19b expression and lead to a decrease in PITX1 expression in melanoma cells. In addition, our present data showed that *PITX1* inhibits *SOX10* expression. It is therefore likely that a negative feedback relationship exists for the regulation of *PITX1* and miR-19b via* SOX10* expression in melanoma cells. Further study involving a detailed analysis of the regulation of miR-19b and *SOX10* transcription may contribute greatly to the discovery of a novel melanoma inhibition pathway.

We and the other researchers showed that *SAMMSON* expression is positively correlated with the expression level of *SOX10*. In this study, *SAMMSON* expression level was less reduced in SKMEL28 compared with A2058 by the overexpression of PITX1, whereas *SOX10* was more efficiently abolished in SKMEL28 compared with A2058 (Fig. 2B,D). Furthermore, another group indicated that sumoylation at K55 in *SOX10* is important for its transcriptional activity toward *SAMMSON* under the treatment of BRAF inhibitor^31^. Therefore, it is possible that the amount of sumoylated *SOX10* protein was more reduced in A2058 compared with in SKMEL28 under the *PITX1* overexpression condition.

We previous study showed that *PITX1* induced apoptosis in human breast cancer MCF-7 cells as a *p53* activator, and forced expression of *PITX1* resulted in *p53*-independent apoptosis in human osteosarcoma MG-63 cells via an unknown mechanism^10^. In addition, *PITX1* expression is downregulated in osteosarcoma tissues and is correlated with the patient survival rate^32^. In our study, overexpression of *PITX1* resulted in apoptosis both in human melanoma A2058 and SKMEL28 cells that carry a dysfunctional *p53* mutation^33^. Therefore, our results indicate that the PITX1-SOX9 signaling pathway could contribute to the regulation of *p53*-independent apoptosis to prevent development of cancers including melanoma and osteosarcoma.

We previously identified *PITX1* as one of the regulatory factors for telomerase activity that controls *hTERT* transactivation, and as a novel tumor suppressor gene^11^. Telomerase activity, which is detected in most cancer cells, contributes to cell immortalization via elongation of telomeres. Mutation of the *hTERT* promoter is reported to occur in over 70% of human melanoma tissues. Mutation hotspots were discovered in the promoter region (− 146C > T and − 124C > T) and are known to generate a new motif for transcription factors, which has been linked to ectopic *hTERT* reactivation^34^. We showed that over-expression of *PITX1* suppressed *hTERT* mRNA transcription using A2058 cells with an *hTERT* promoter mutation (− 124C > T)^10^. This result indicated that *PITX1* has the ability to suppress *hTERT* expression regardless of the mutation status of the *hTERT* promoter. *PITX1* thereby plays a crucial role in regulating oncogenic signaling pathways, such as telomerase activity and *SOX-SAMMSON* transcription, suggesting that control of *PITX1* may strongly contribute to a novel therapeutic strategy to target melanoma.

*BRAF* mutations have been identified in 40–50% of melanoma patients^4^. *BRAF* inhibitors (e.g., vemurafenib and dabrafenib) contribute to improvements in the clinical status of these patients. Unfortunately, resistance to these agents typically occurs 6–8 months after initiation of therapy in the vast majority of patients treated with vemurafenib and dabrafenib^5^. Therefore, identification of new targets for melanoma therapy is important to overcome the issue of resistance to drug therapy. *SAMMSON* silencing reduced cell proliferation and induced apoptosis independently of the *BRAF*, *NRAS*, or *p53* mutation status in melanoma cells. Importantly, melanoma cells showing resistance to *BRAF* inhibitors were also sensitive to *SAMMSON* silencing. *SAMMSON*, which is one of the novel candidate targets of melanoma therapy^24^, is regulated by the *SOX10* gene. The *SOX10* binding consensus sequence is present upstream of the *SAMMSON* gene loci; moreover, knockdown of *SOX10* led to a reduction in *SAMMSON* expression^24^. In our study, overexpression of *PITX1*, which was correlated with suppression of *SOX10* expression, reduced *SAMMSON* expression. On the other hand, sorafenib is a kinase inhibitor that acts on many protein kinases, such as *VEGF*, *PDGFR*, and *RAF,* and is also reported to up-regulate *PITX1* by inhibiting the phosphatase activity of *PTP1B*, which promotes proteasomal degradation of *PITX1*^35^. Restoration of *PITX1* by sorafenib treatment resulted in inhibition of *RAS* activity in hepatocellular carcinoma (HCC) cell lines. Additionally, inhibition of *PTP1B* phosphatase activity was accompanied by up-regulation of *PITX1* expression in colon cancer cell lines by the receptor tyrosine kinase inhibitor regorafenib^36^. The Expression Atlas web tool showed that *PTP1B* expression is positive in melanoma cell lines as well as in colon and HCC cell lines (https://www.ebi.ac.uk/gxa/home). These data suggest the possibility that sorafenib and regorafenib could up-regulate *PITX1* in melanoma cells, which may affect the *SOX9*/*SOX10* signaling via restoration of *PITX1* expression. Thus, more detailed experiments using combinations of *BRAF* inhibitors and *PITX1* inducing drugs for melanoma treatment will lead to the development of anticancer agents through novel targets.

## Materials and methods

### Cell culture

Cell culture was performed as described previously^37^. A2058 cells were obtained from the Japanese Collection of Research Bioresources Cell Bank (Osaka, Japan). SKMEL28 cells were obtained from the Cell Resource Center for Biomedical Research, Institute of Development, Aging and Cancer, Tohoku University, Japan. A2058 and SKMEL28 cells were cultured in Dulbecco's Modified Eagle Medium (DMEM; Sigma, St. Louis, MO, USA) supplemented with 10% fetal bovine serum (FBS, HyClone, Logan, UT, USA). WM3211 cells were obtained from the Rockland Immunochemicals Inc (Limerick, PA, USA) and maintained in a culture medium consisting of 80% MCDB153 (Sigma) with 20% Leibovitz’s L-15 (Sigma), 2% FBS (HyClone), and 1.68-mM CaCl_2_ (Sigma). All cells were cultured at 37 °C in a humidified incubator under 5% CO_2_. FLAG-PITX1 stable expression A2058 clones were maintained in DMEM supplemented with 10% FBS and G418 (300 μg/ml; Calbiochem, La Jolla, CA, USA). All cell lines were confirmed to be mycoplasma-free using a MycoAlert mycoplasma detection kit (Lonza, Walkersville, MD, USA) and were not passaged more than 20 times from the validated stocks. Fluorescent images were obtained using a KEYENCE BZ-X710 microscope (Keyence, Osaka, Japan) using an objective with a 40 × magnification.

### Cell proliferation assay

Cell proliferation assay was performed as described previously^29^. Cells (2.5 × 10^4^ cells/ml) were seeded in a 6 cm^2^ culture dish. Cells were counted each day and the average cell number in three dishes was quantified using a Coulter Counter Z2 (Beckman Coulter, Woerden, Netherlands).

### Analysis of apoptosis

Analysis of apoptosis was performed as described previously^37^. Apoptotic cells were measured by Annexin V staining using an APC Annexin V apoptosis detection kit with 7-AAD according to the manufacturer's (BioLegend, San Diego, CA, USA) instructions. Annexin V positive but 7-AAD negative (early apoptotic cells) and Annexin V positive and 7-AAD positive (late-stage apoptosis) cells were determined using a Gallios flow cytometer and analyzed using Kaluza software (Beckman Coulter, Brea, CA, USA).

### ATP assay

The ATP assay was performed using CellTiter-Glo 2.0 (Promega, Madison, WI, USA), according to the manufacturer’s protocol.

### Chromatin immunoprecipitation sequence analysis

We performed chromatin immunoprecipitation (ChIP) as described in Ref.^38^. A2058 cells were cross-linked for 10 min by 1% formaldehyde and fragmented by a sonicator (UD-211; TOMY SEIKO, Tokyo, Japan). The samples were immunoprecipitated with 4 μg of antibodies against H3K27ac (05-1334, Merck Millipore, Billerica, MA, USA). We used protein A-sepharose beads and G-sepharose beads (GE Healthcare, Barrington, Ill, USA) to immunoprecipitate the fragments. Chromatin immunoprecipitation sequence (ChIP-Seq) libraries were made using a KAPA Hyper Prep kit (Roche, Wilmington, MA, US) with 1 ng of ChIP DNA. Libraries were size-selected prior to PCR amplification using AMPure XP beads (Beckman Coulter, Brea, CA, USA). Multiplexed libraries were run on an Illumina HiSeq 2500 genome sequencer (Illumina Inc. San Diego, CA, USA) using the 50-base pair single read method. The nucleotide sequence data reported are available in the DDBJ Sequenced Read Archive under the accession numbers DRX230605 and DRX230606.

### Western blotting analysis

Western blotting was performed as described previously^37^. Membranes were blotted with rabbit polyclonal antibody against human PITX1 antigen (ab70273, 1:2,000; Abcam, Cambridge, MA, UK), rabbit monoclonal antibody against human SOX9 antigen (ab185966, 1:2000; Abcam), rabbit monoclonal antibody against human SOX10 antigen (ab155279, 1:2000; Abcam), mouse monoclonal antibody against FLAG antigen (F1804, 1:2000; Sigma), or with polyclonal antibody against α-tubulin (PM054-7, 1:5000; MBL, Tokyo, Japan) and the appropriate standard peroxidase-labeled anti-mouse IgG and anti-rabbit IgG secondary antibodies, according to the manufacturer's instructions (GE Healthcare, Piscataway, NJ, USA). Immunoreactive bands were visualized using the ECL detection system (Pierce, Rockford, IL, USA).

### qRT-PCR

RNA isolation and reverse transcriptase (RT)-PCR were performed as described previously^37^. mRNA expression of SAMMSON was analyzed using specific primers: SAMMSON: forward; 5′-CCTCTAGATGTGTAAGGGTAGT, reverse; 5′-TTGAGTTGCATAGTTGAGGAA. cDNA was amplified using an Applied Biosystems StepOne thermal cycler system and SYBR green PCR kit (Foster City, CA, USA). mRNA levels were normalized against GAPDH mRNA (PCR primers: forward; 5′-AGCCACATCGCTCAGACAC, reverse; 5′-GCCCAATACGACCAAATCC).

### ChIP assay

The chromatin immunoprecipitation (ChIP) assay was performed as described previously^37^. The ChIP assay was performed with a SimpleChIP Plus Sonication Chromatin IP kit (Cell Signaling Technology (CST), Danvers, MA, USA) according to the manufacturer's protocol. Briefly, to cross-link the DNA in chromatin to histones, the cells were incubated in 1% formaldehyde for 10 min at 37 °C. After being washed with cold phosphate-buffered saline (PBS) containing protease inhibitors (CST), the cells were resuspended in cell lysis buffer (CST) containing protease inhibitors. DNA then was broken into 100- to 300-bp fragments using a Covaris S220 sonicator (Woburn, MA, USA). The remainder of the sample was immunoprecipitated using anti-PITX1 antibody (ab70273, Abcam) or anti-rabbit IgG antibody (#2729, CST) for 16 h at 4 °C. Protein G magnetic Beads (CST) were then used to collect the immunoprecipitated complexes, which were eluted using elution buffer (CST) after extensive washing. Cross-linking was then reversed by the addition of 5 M NaCl, which was followed by protease K treatment for 12 h at 65 °C. DNA was recovered using DNA purification columns (CSTs), and was used as a template for PCR amplification of the region of the PITX1 binding sites in the SOX9 promoter. The forward and reverse PCR primers used were: forward, 5′-TTGCAAAAGCGCAGCAGAAT-3′ and reverse, 5′-GTTTTGGTGACTCAACGCCC-3′. DNA was amplified using an Applied Biosystems StepOne thermal cycler system and a SYBR green PCR kit.

### Plasmid construction

FLAG-tagged *PITX1* expression plasmids were generated as described previously^29^. FLAG-tagged *PITX1* expression lentivirus plasmid pFLAG-PITX1 was constructed by PCR amplification of PITX1 cDNA without the 3′UTR region from genomic DNA using KOD plus DNA polymerase (TOYOBO, Tokyo, Japan) and the following primer sequences: forward primer: 5′-GCTCTAGAATGGACGCCTTCAAGGGGGGCATGAGCCTG, reverse primer: 5′-CGGGATCCTCAGCTGTTGTACTGGCACGCGTTGAGGCC, and was inserted into the XbaI/BamHI digested pLVSIN-EF1α-IRES-ZesGreen1 vector (Takara, Shiga, Japan). The pLVSIN-EF1α-IRES-ZesGreen1 vector was used as a control. Production of lentivirus was performed using a Lentiviral High Titer Packaging Mix (Takara) according to the manufacturer’s protocol.

Various lengths of the SOX9 promoter region (shown in Fig. 3A), which included the transcription start site, were PCR amplified from genomic DNA using KOD plus DNA polymerase (TOYOBO) and inserted into the Acc65I/BglII-digested luciferase (Luc) reporter vector pGL3-basic (Promega). The primer sequence is as follows: for SOX9 pro -1100; forward primer: 5′-GAGGTACCGTGGAGCGTTTTGTCTGCGGTGGTG, reverse primer: 5′-GGAAGATCTTGAAACTGGCGAGTCTCCGCGCCAC, for SOX9 pro − 540; forward primer: 5′-GAGGTACCAACATTTGCTTCAAAAGACTATTTC, reverse primer: 5′-GGAAGATCTTGAAACTGGCGAGTCTCCGCGCCAC, for SOX9 pro − 486; forward primer: 5′-GGTACCGCTACGCATTAAGAAGCGGCTGCTT, reverse primer: 5′-GGAAGATCTTGAAACTGGCGAGTCTCCGCGCCAC.

A PCR-based site-directed mutagenesis kit (Toyobo) was used to generate mutations in a number of nucleotides in the RE site of the SOX9 promoter (shown in Fig. 3D). The PCR primers used were: RE1; forward primer: 5′-GGTACCTATTTATATGGATTA TTACGGAGGA, reverse primer: 5′-ACATGCTCGGGTTCGCCGGGGCTGG, RE2; forward primer: 5′-GGTACCTTACGGAGGAACAGCGGGCGTTGAG, reverse primer: 5′-ATATAAATAGATTAACATGCTCGGG, RE3; forward primer: 5′-GGTACCCAGGCAGGCAGGCTCGCTCCAGGCG, reverse primer: 5′-GGTACCAGTCTTTTGAAGCAAATGTTTTGGT. The sequences of all plasmids were confirmed by DNA sequencing.

### Luciferase assay

Luciferase assay was performed as described previously^11^. The cells were plated in 12-well plates 24 h before transfection. *SOX9* promoter reporter plasmids and *PITX1* expression plasmids were co-transfected using Lipofectamine LTX reagent (Invitrogen) according to the manufacturer’s protocol. pGL4.70-renilla (Promega) was co-transfected as an internal control. The cells were lysed 48 h after transfection and subjected to a luciferase assay using the Picagene dual Sea Pansy bioluminescence kit (Toyo Ink, Tokyo, Japan) according to standard protocols. All experiments were performed in at least triplicate. Luciferase activity was calculated as the activity of the reporter constructs compared to the *Renilla* activity.

### Expression analysis of human melanoma tissue

Human melanoma tissue mRNA dataset GSE15605, GSE46517 and TCGA-SKCM was downloaded from the GEO (http://www.ncbi.nlm.nih.gov/geo/) and TCGA database (https://portal.gdc.cancer.gov/). GSE15605 included twelve metastatic tumor samples from melanoma patients and sixteen normal skin samples without melanoma as controls, and GSE46517 included seventy metastatic tumor samples from melanoma patients and eight normal skin samples without melanoma as controls. Relative mRNA expression level in the GEO data set was normalized to GAPDH. TCGA-SKCM included 103 primary and 368 metastatic tumor samples from patients with melanoma. Relative mRNA expression level in the TCGA data set was analyzed using the UCSC Xena browser (https://xenabrowser.net/). We used only the mRNA dataset from GEO and TCGA. We have not used any human tissue samples in this study.

### Xenograft analysis

All animal experiments were approved by the Institutional Animal Care and Use Committee of Tottori University (Approval number: 19-Y-42). The mice were maintained under specific pathogen-free conditions with a 12-h ligh–dark cycle. Four-week-old female BALB/c-nu/nu (BALB/cSlc-nu/nu) mice were obtained from SLC Japan (Japan, Shizuoka). The animals were injected subcutaneously in the right and left flank regions with 1.0 × 10^7^ A2058 cells expressing FLAG-PITX1 and FLAG-control suspended in 100 ml (PBS). All experiments and methods were performed in accordance with the Animal Research: Reporting of In Vivo Experiments (ARRIVE) guidelines^39^. We declared that all methods were carried out in accordance with the relevant guidelines and regulations.

### Immunostaining

Luciferase assay was performed as described previously^11,16^. Dewaxed paraffin sections were used for immunofluorescent staining. The sections were incubated with rabbit polyclonal antibody raised against PITX1 (ab70273, 1:2000; Abcam), SOX9 (ab185966, 1:500; Abcam), SOX10 (ab155279, 1:10,000; Abcam), Ki-67 (pre-diluted, Nichirei, Tokyo, Japan) or cleaved Caspase3 (#9664, 1:400; Cell Signaling Technology) at 4 °C overnight. After being washed in T-TBS, the sections were incubated with secondary Alexa488-conjugated anti-rabbit IgG antibody (#8890, 1:500, Cell Signaling Technology) for 1 h at room temperature. After further washing, coverslips were placed on the glass slides using a water-soluble mounting medium. The slides were observed using fluorescence microscopy. Images were captured using an AxioImagerZ2 fluorescence microscope (Carl Zeiss GmbH, Jena, Germany) with a 40 × objective.

### Graphs

Cell growth curves, bar graphs, box plots, and scatter plots were created using Excel (Microsoft Corporation, Redmond, WA, USA). Cell count images of Annexin V-positive cells were created using Kaluza software (Beckman Coulter).

### Statistics

Statistics was performed as described previously^29^. Data from more than three separate experiments are presented as means ± S.D. Significance was established at P-values less than 0.05 using an unpaired two-tailed Student’s t-test. The relationship between the expression levels of genes was calculated by Pearson’s correlation coefficient (r).

## Supplementary Information

Supplementary Information.

## Data Availability

All data is provided in the manuscript.
