# Supplementary material for: PITX1 inhibits the growth and proliferation of melanoma cells through regulation of SOX family genes
Source: Sci Rep. 2021 Sep 15;11:18405. doi: 10.1038/s41598-021-97791-6 (PMC8443576; doi:10.1038/s41598-021-97791-6)
Supplement: Supplementary file 1 — Supplementary Information. [file 41598_2021_97791_MOESM1_ESM.docx]

**Supplementary Information**

***PITX1* inhibits the growth and proliferation of melanoma cells through regulation of *SOX* family genes**

Takahito Ohira, Suguru Nakagawa, Jumpei Takeshita, Hiroyuki Aburatani, Hiroyuki Kugoh


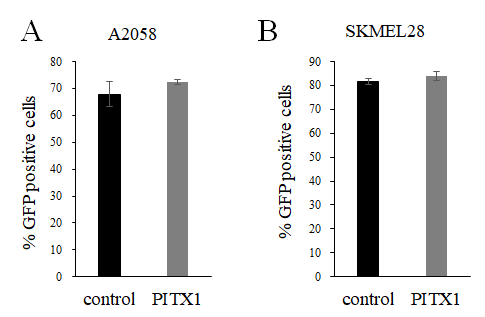


**Supplementary Fig S1. Transduction efficiency of lentivirus vector in melanoma cell lines.** (A) A2058. (B) SKMEL28. GFP positive cells were quantified by flow cytometry. These bar graphs were created by Excel.


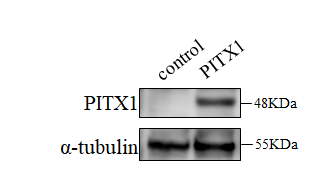


**Supplementary Fig S2. Expression analysis of PITX1 stably over-expressing cells.** Western blotting of the PITX1 protein level in PITX1 stably expressing A2058 cells. The expression levels of PITX1 were normalized to the levels of α-tubulin. Cropped blots were used in this figure. Original full-length blots are presented in Supplementary Fig. S8.


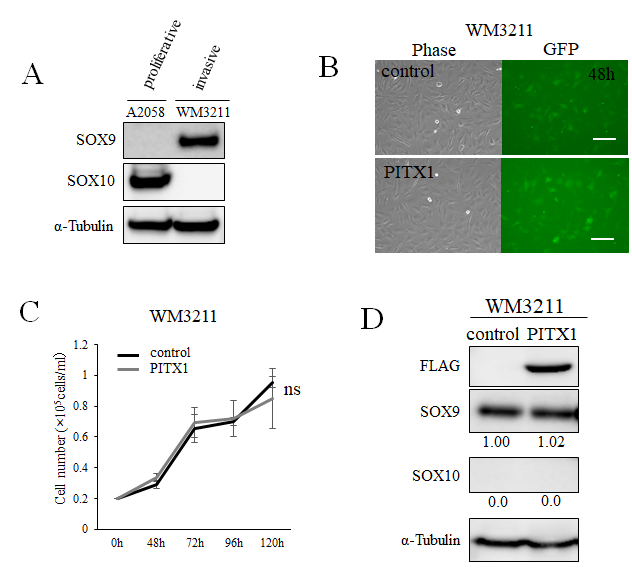


**Supplementary Fig S3. Overexpression analysis of *PITX1* in invasive type melanoma cell lines. A**. Western blotting of the SOX9 and SOX10 protein level in A2058 and WM3211 cells. The expression levels of proteins were normalized to the levels of α-tubulin. Cropped blots were used in this figure. Original full-length blots are presented in Supplementary Fig. S8. **B**. WM3211 cells were infected with control and *PITX1* expression lentivirus vectors. Infection efficiency was monitored after 48 hr by fluorescence analysis of vector-encoded GFP (right panels). Phase contrast images are shown at left. Scale bars: 100 µm. **C**. Number of *PITX1* transfected cells and control cells over 5 days. Bars correspond to means ± S.D. for three independent experiments (ns: not significant). This image of growth curve was created using Excel. **D**. Western blotting analysis of the protein level of PITX1 (Flag tagged), SOX9 and SOX10 in WM3211 cells at 48 hr after infection with PITX1 expressing or control lentivirus vector. The expression levels of SOX9 and SOX10 were normalized to α-tubulin levels. Cropped blots were used in this figure. Original full-length blots are presented in Supplementary Fig. S8.

(-720) 5’-

CTCCTGCGTGGCCACGGCCGCCGCTGCCAACCTTCGCGGGGACTTAGCTTTGCTTTCCATTGACTCCCTTTGCAAAAGCGCAGCAGAATCCTGACCAGCCGCACCAGCCCCGGCGAACCCGAGCATGT**TAATC**TATTTATAT**GGATTA**TTACGGAGGAACAGCGGGCGTTGAGTCACCAAAACATTTGCTTCAAAAGACT**ATTTCTAAGCACTTTTG**CAGGCAGGCAGGCTCGCTCCAGGCGCGTAAACTCGGCTACGCATTAAGAAGCGGCTGCTTTTCGAATACTGCAAACTCCAGCTAAGTCCCCGGTGCCGCGGAGAGAGCAGTGAAAAGAAATGTCGGAGGTGGGGGTAGATCCTAGTCTAGACACACACACTTGCG

-3’ (-340)

**RE1: TAATC**

**RE2: GGATTA**

**RE3: ATTTCTAAGCACTTTTG**

**Supplementary Fig. S4. PITX1 regulatory elements of SOX9 promoter region.** The sequences within the *SOX9* promoter. There are three RE sites in the *SOX9* promoter. Under line shows RE sites.


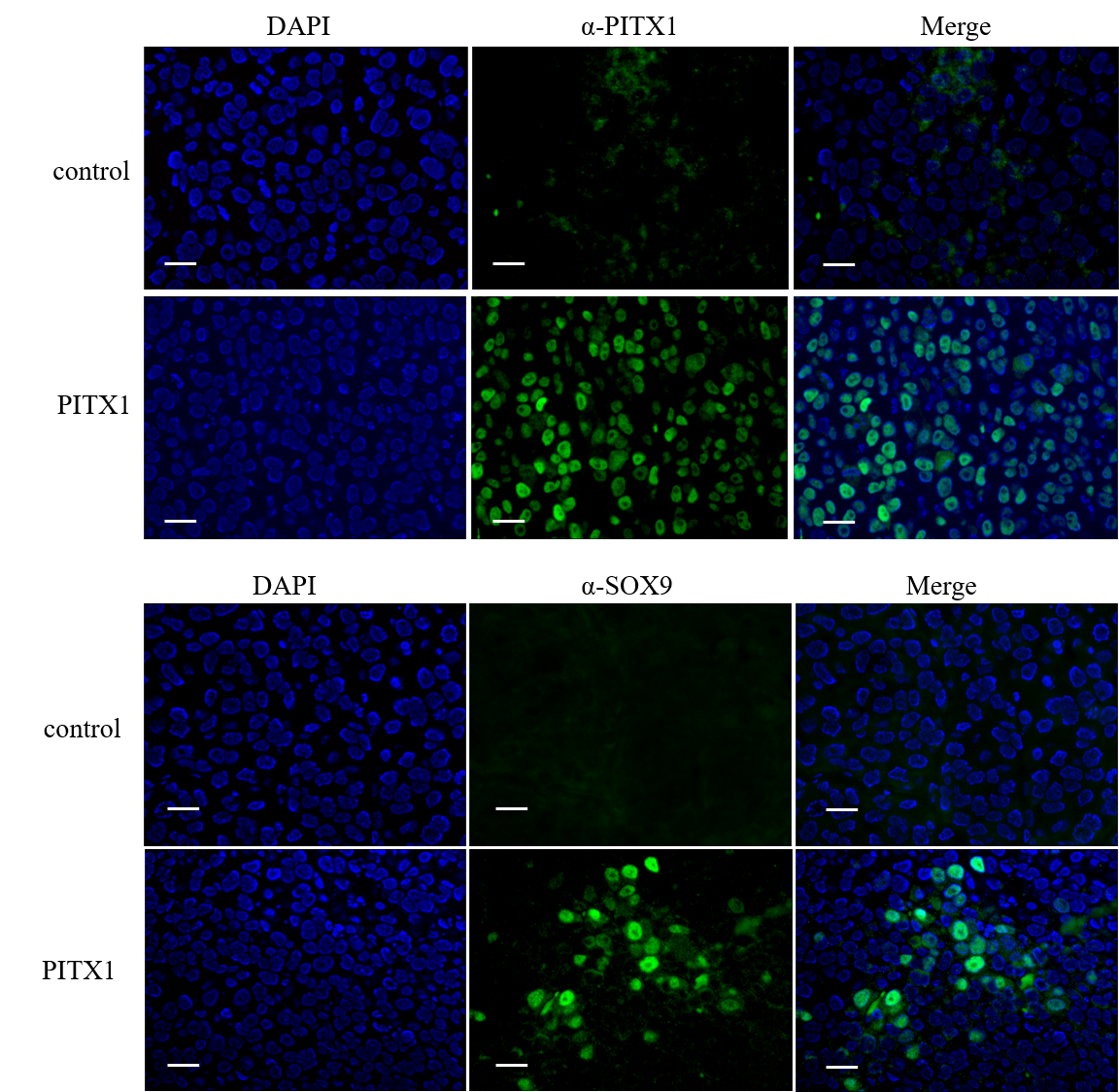

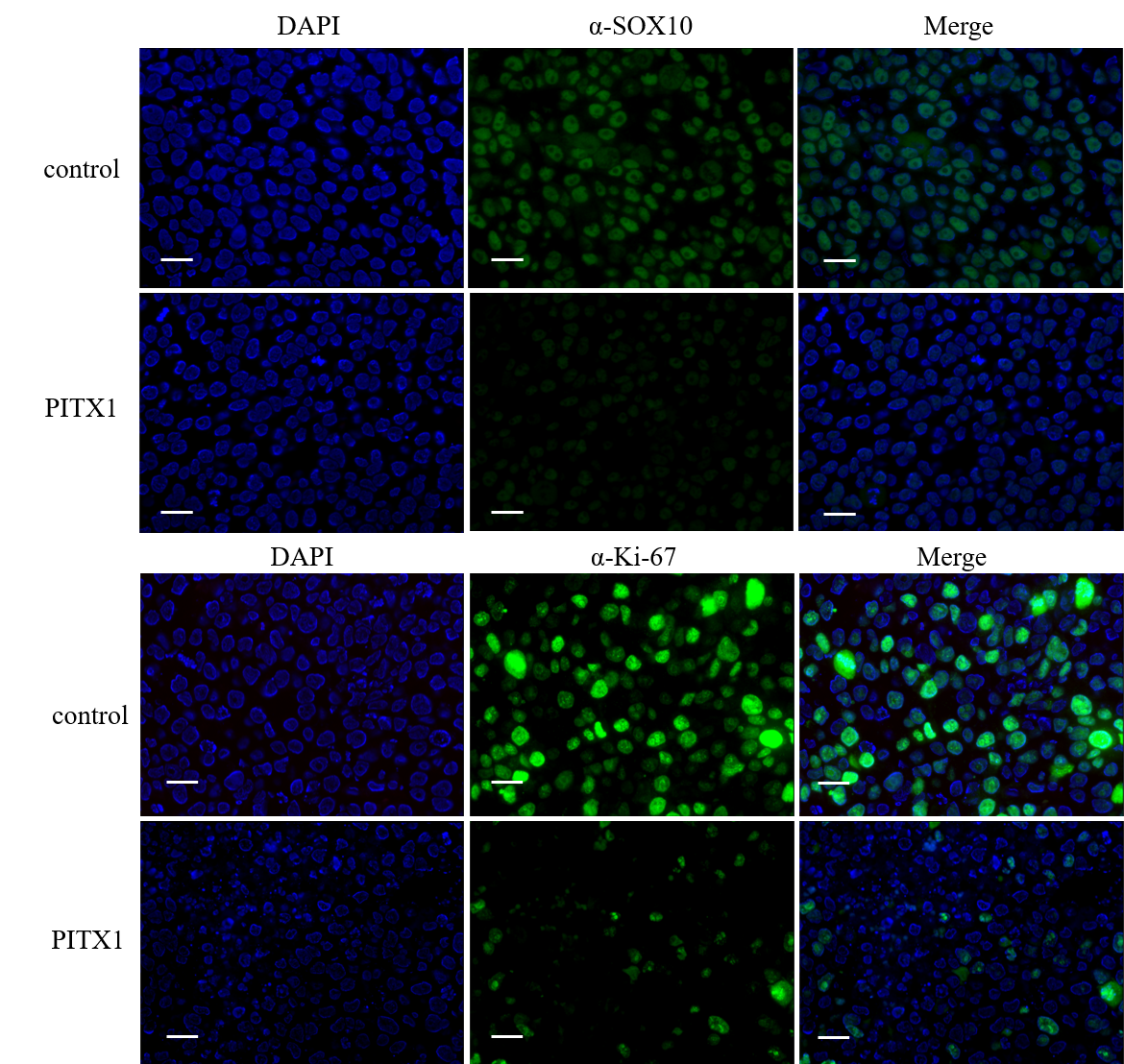

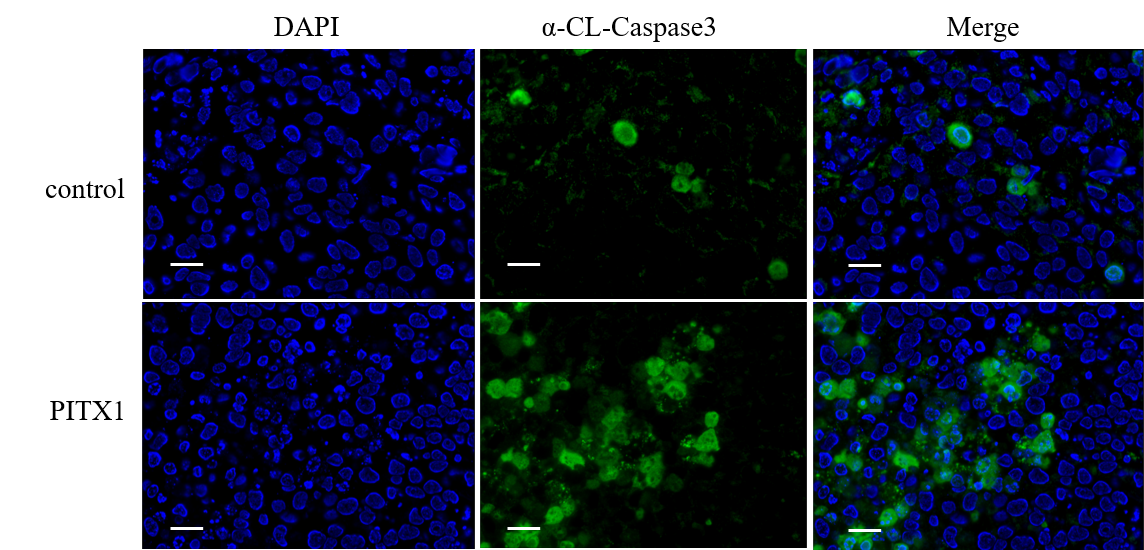


**Supplementary Fig. S4.** Individual images related to Figure 5D-H.


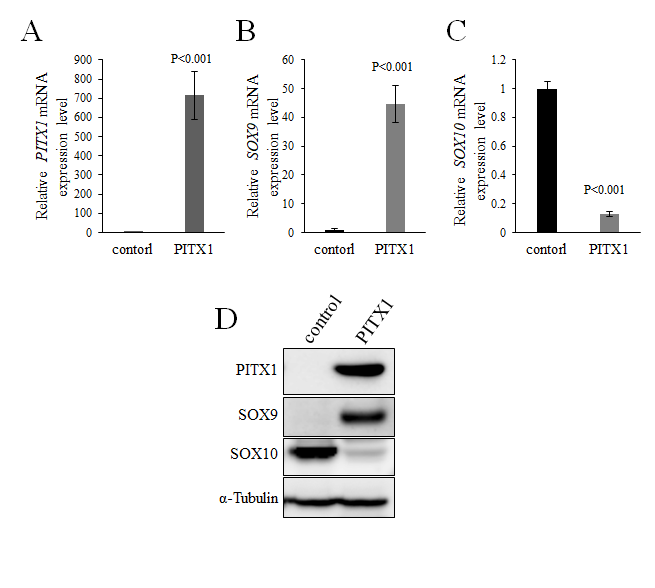


**Supplementary Fig. S6.** Results of qRT-PCR and WB related to Figure 5D-F. Expression in the vector control cells was arbitrarily set at 1. *GAPDH* mRNA expression was used as the internal control. Data are presented as means ± S.D. of three independent experiments (P<0.001). These bar graphs were created using Excel. Cropped blots were used in this figure. Original full-length blots are presented in Supplementary Fig. S8.


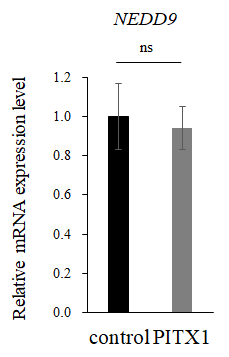


**Supplementary Fig. S7.** qRT-PCR analysis of relative *NEDD9* RNA expression levels in *PITX1* and control lentivirus vector infected A2058 cells. Expression in the vector control cells was arbitrarily set at 1. *GAPDH* mRNA expression was used as the internal control. Data are presented as means ± S.D. of three independent experiments (ns: not significant). This bar graphs was created using Excel.


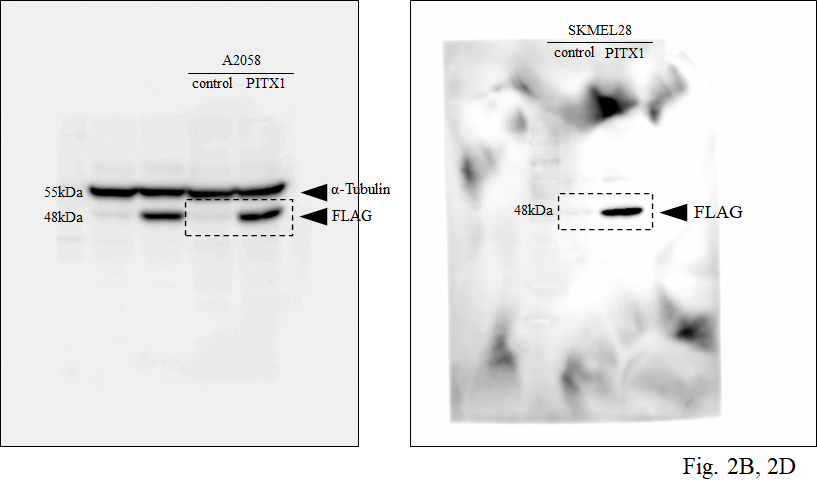

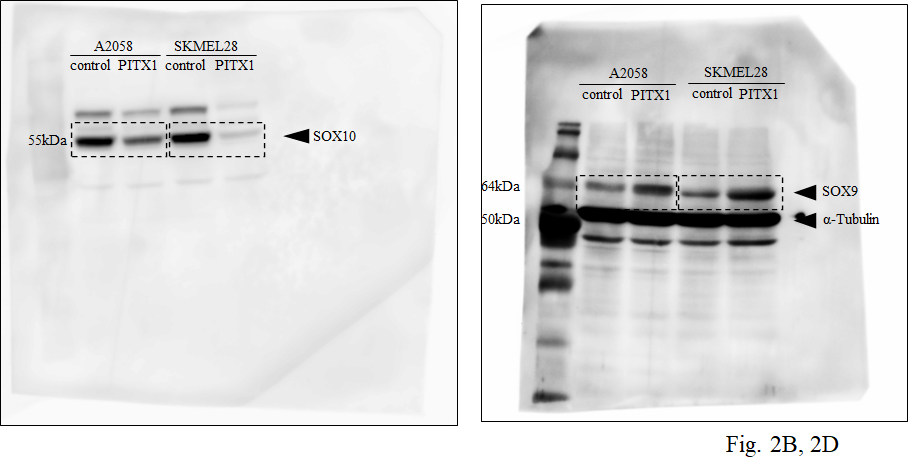

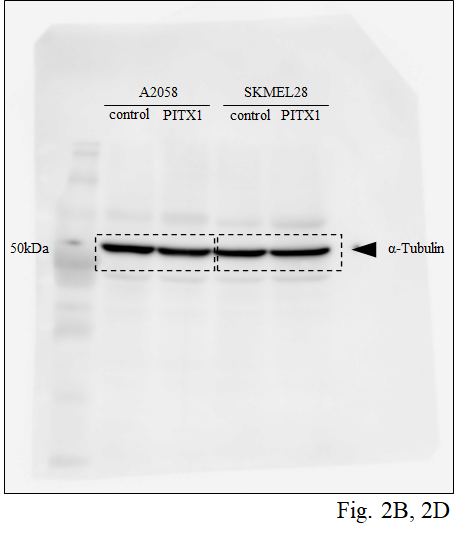


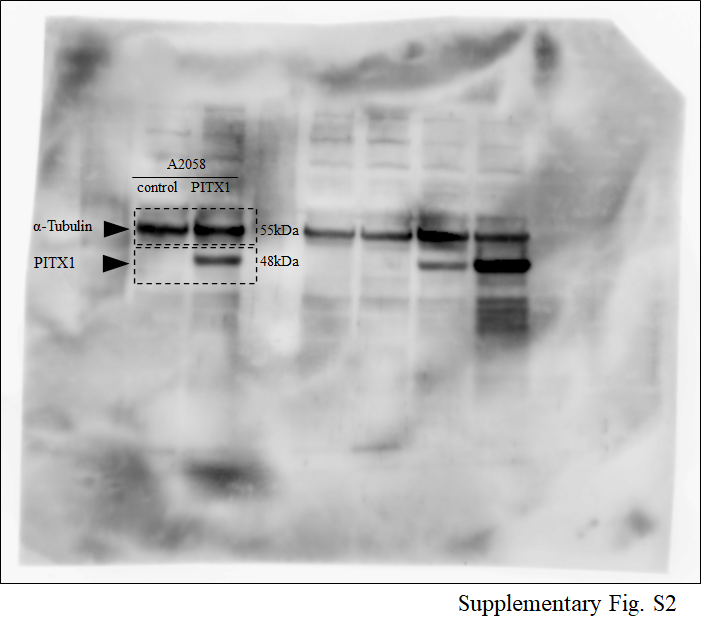

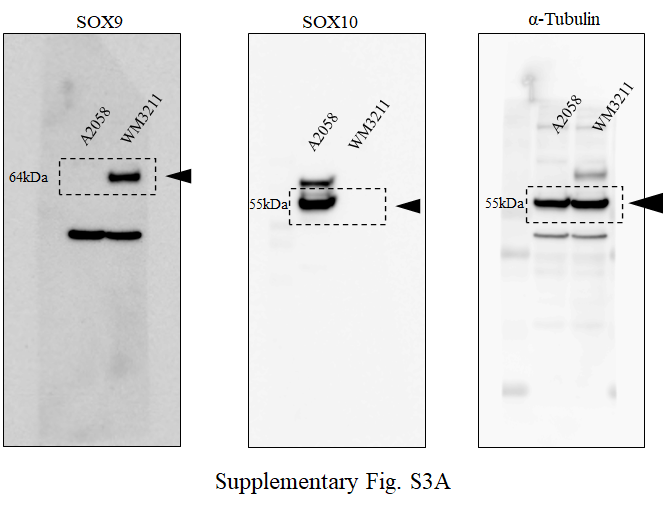

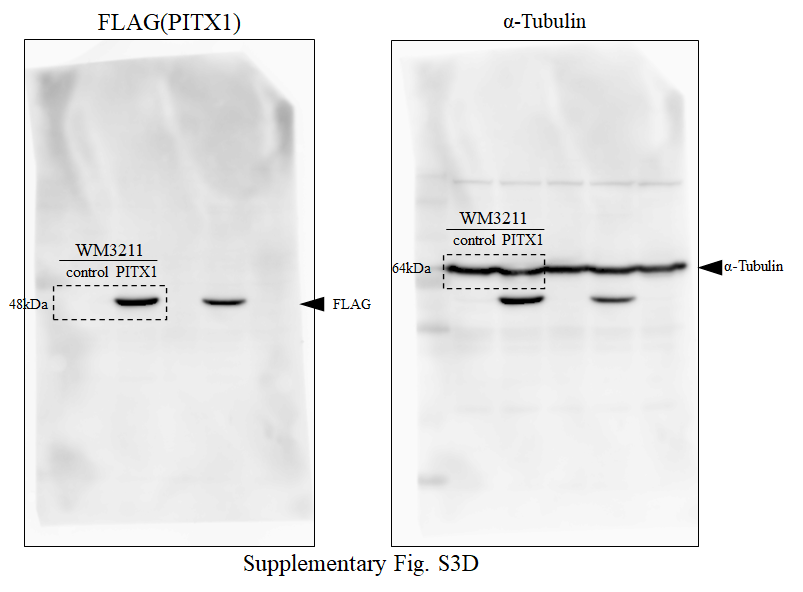


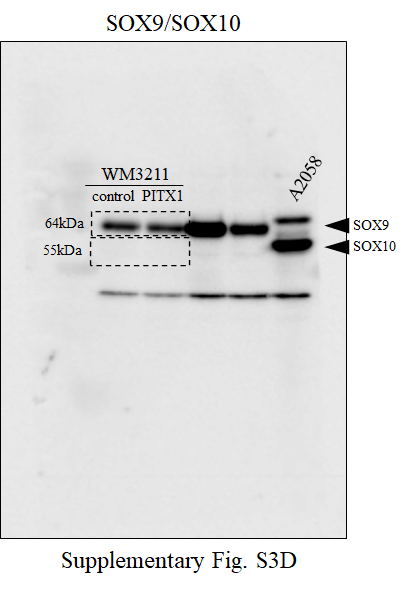

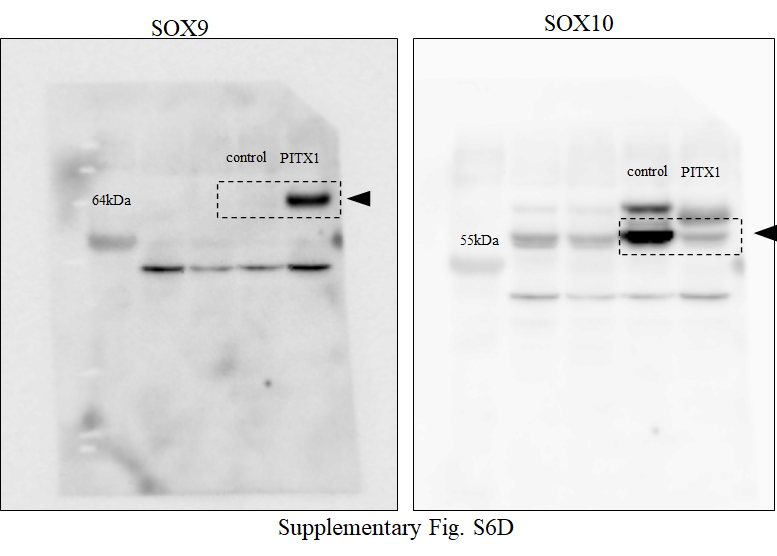

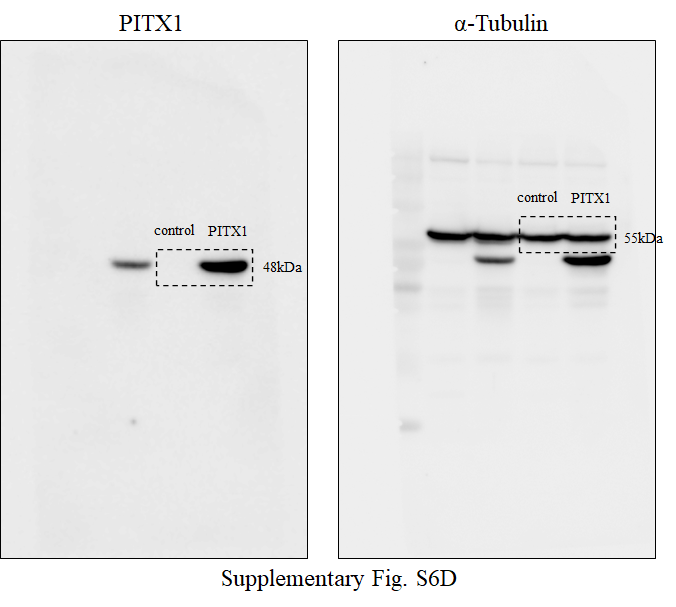


**Supplementary Fig. S8.** Unprocessed images of immunoblots. Unprocessed images of scanned immunoblots as shown in Fig. 2B, 2D and Supplementary Fig. S2, S3A, S3D and S6D are provided. Regions that are presented in the main figures are outlined with a dashed black rectangle.
